# Cyclosporine and Herbal Supplement Interactions

**DOI:** 10.1155/2014/145325

**Published:** 2014-01-12

**Authors:** D. Colombo, L. Lunardon, G. Bellia

**Affiliations:** ^1^Novartis Farma S.p.A., Origgio, Varese, Italy; ^2^Marchesi Hospital, Inzago, Milan, Italy; ^3^Department of Pathophysiology and Transplantation, University of Milan, I.R.C.C.S. Foundation, Cà Granda Ospedale Maggiore Policlinico, Milan, Italy

## Abstract

Cyclosporine (CyA) is a well-known immunosuppressant with a narrow therapeutic window. Its bioavailability is affected by many other traditional drugs and herbal extracts. Cytochrome P-450 isoenzymes CYP3A4 and CYP3A5 and protein P-glycoprotein (P-gp) are involved in CyA bioavailability. Interactions of CyA with herbal extracts are not well known, but, given their increased concomitant use, it is important to know which extracts, many of which are commonly self-prescribed, can affect CyA blood concentrations. Decreased CyA blood concentration has been shown with St John's wort in case reports and, in vivo animal studies, with ginger, liquorice, scutellariae radix, and quercetin. Increased CyA concentration has been reported in patients with grapefruit juice, chamomile, or berberine, and with cannabidiol or resveratrol in animal studies. Effects of *Echinacea* and *Serenoa repens* on CyA levels have not been shown consistently, but concomitant use should be avoided. Although findings from animal studies cannot be directly translated into humans, avoiding concomitant use of herbal extracts is prudent until human clinical studies have ruled out any possible interaction. Clinicians should interview their patients carefully about their use of herbal supplements before CyA administration, and those receiving CyA should be warned about possible interactions between herbal preparations and CyA.

## 1. Introduction

In 1972, cyclosporine (CyA) was discovered and identified as a new antifungal drug with unexpected immunologic characteristics. Subsequently, the effect of CyA on lymphocyte activation was demonstrated [1]. Since this discovery, CyA was introduced as a new immunosuppressant therapy and, in the last three decades, has been widely used in transplant recipients to prevent rejection and in the treatment of autoimmune diseases, with successful results. CyA is also widely used in the treatment of psoriasis, atopic dermatitis, and other dermatological disorders. CyA is a lipophilic cyclic polypeptide that selectively inhibits the adaptive immune response. This activity is exerted through CyA binding to cyclophilin, inhibition of calcineurin, and nuclear factor transcription, with a subsequent alteration in T-cell activation. The lipophilic nature of CyA required the development of a triphasic microemulsion preparation with higher hydrophilicity to reduce the interindividual variability in intestinal absorption.

CyA is metabolized in the liver and small intestine by isoenzymes CYP3A4 and CYP3A5 of the cytochrome P-450 family [2]. Protein P-glycoprotein (P-gp), an efflux pump, is further involved in CyA bioavailability, as CyA is a P-gp substrate and is therefore pumped out of cells by P-gp [3]. CYP3A and P-gp are colocalized in enterocytes (small intestine), hepatocytes, and renal proximal tubular cells [4].

CyA has a narrow therapeutic window; for example, plasma concentrations should be maintained between 250 and 400 ng/mL in transplant recipients [5]. Many factors can affect CyA metabolism and bioavailability and clinicians and patients need to be well informed to avoid inadvertently altering CyA blood concentrations. Most differences in CyA bioavailability are due to food ingestion, changes in gastric motility, diarrhea, diabetes, and CYP3A5 genetic polymorphism. In addition, a major cause of reduced or increased CyA metabolism is the coadministration of drugs and other substances that are able to inhibit or induce the activity of cytochrome P-450 enzyme or P-gp or their effect on CyA.

In recent years, an increased consumption of herbal medicines worldwide has increased the need to understand potential interactions between these compounds and traditional drugs, especially those with a narrow therapeutic window, such as CyA. About 300 species of plants are commonly used in medicine and some of them interfere with the function of cytochrome P-450 and P-gp. These include St John's wort (SJW), grapefruit juice, ginger, chamomile, scutellariae radix, and quercetin. In view of the increasing use of herbal remedies worldwide, the purpose of this paper is to provide updated information on herbal-CyA interactions. A summary of commonly used herbal extracts, the outcome of herb-CyA interactions, and the relevant mechanisms are shown in Table 1.

## 2. St John's Wort

SJW is a herbal medicine extracted from *Hypericum* and utilized as an antidepressant drug. SJW contains more than 20 components. Hyperforin and hypericin are the two major constitutes [6]. Hypericin regulates P-gp, while hyperforin is able to induce both CYP3A4, CYP2B6, and P-gp. The induction of both intestinal and hepatic CYP3A4 and drug transporter P-gp is due to the activation of the pregnane X receptor and the subsequent expression of a range of genes [7]. Since CyA is metabolized via CYP3A4 and P-gp, it is likely that SJW may alter CyA bioavailability. In an open label study investigating the effects of SJW on CyA metabolism in 11 renal transplant patients receiving 600 mg SJW plus the regular dosage of CyA for 2 weeks, SJW administration resulted in clinically relevant decreases in CyA concentration after 3 days in all treated patients, and 41–46% reduction in plasma CyA concentration after 2 weeks of oral SJW intake [8].

Various case reports in the literature have described failed CyA treatment due to taking SJW. One case report described two patients who both received a kidney transplant and maintenance immunosuppressant therapy with corticosteroids and CyA ± mycophenolate mofetil. Both patients experienced a reduction in CyA concentration below the target level (200 ng/mL) and experienced acute rejection. Both patients were self-medicated with SJW. Plasma CyA concentration increased dramatically after discontinuing the herbal medicine [9]. Another report described a kidney transplant patient with a stable CyA blood concentration for several months followed by a rapid decrease after 2 weeks of SJW therapy. In this case, it took 1 month without SJW for CyA levels to normalize [10]. Two heart transplant patients experienced acute rejection while taking CyA, azathioprine, and corticosteroids. The rejection was attributed in both cases to SJW and the subsequent decrease of CyA plasma concentration. This was confirmed when SJW was discontinued and CyA returned to therapeutic concentrations. Taking all of these studies and reports into consideration, SJW must be avoided during CyA treatment.

## 3. Grapefruit Juice

In the late 1980s, while investigating a possible interaction between ethanol intake and felodipine, accidentally, grapefruit juice was discovered to dramatically elevate felodipine bioavailability [11]. A subsequent study demonstrated a decrease in intestinal CYP3A4 concentration after grapefruit juice intake, but no changes in hepatic CYP3A4 [12]. As CyA is metabolized by both hepatic and intestinal CYP3A4, grapefruit juice increases CyA bioavailability. Increased CyA concentrations of more than 60% have been reported after intake of grapefruit juice [13, 14]. This increase has been reported only after oral CyA and not intravenous administration of CyA; this is in accordance with the specific inhibition of intestinal but not hepatic CYP3A4.

Various studies have demonstrated the alteration in CyA concentration after grapefruit juice intake in healthy volunteers [15] and transplant patients, both in adults [16] and children [17].

Grapefruit juice must be avoided during oral CyA therapy to maintain therapeutic and nontoxic drug concentrations.

## 4. Ginger

Ginger, a rhizome of the plant *Zingiber officinale*, is widely used as food or medicine. Ginger is commonly used for nausea and vomiting induced by pregnancy, gastrointestinal diseases, and chemotherapy. It also has been demonstrated to have effects on platelet aggregation and tumor growth [18]. A study conducted in rats investigated the role of ginger juice in CyA bioavailability after oral or intravenous administration [18]. The intake of ginger juice significantly decreased the concentration of CyA administered orally but not intravenously. The authors hypothesized that ginger reduces oral CyA bioavailability due to the effects of ginger on gastrointestinal motility [18]. No case reports have been described for CyA and ginger interaction, but their coadministration should be avoided to guarantee optimal CyA blood concentrations.

## 5. Cannabidiol

Cannabidiol is a nonpsychoactive cannabinoid found in marijuana, and it is considered to have medical applications in epilepsy, multiple sclerosis, anxiety, nausea, tumor growth and invasiveness, and schizophrenia [19]. A study conducted in the 1990s on cannabidiol-CyA interaction, utilizing in vivo and in vitro experiments, demonstrated a clear inhibition of mouse and human hepatic microsomal CyA metabolism [20]. Human in vivo studies are not available at the moment, but cannabidiol use, medical or not, should be avoided due to its potential for increasing CyA blood concentration and toxic effects.

## 6. Chamomile

Chamomile is a daisy-like plant utilized in infusions for sedative effects. An in vitro study on human CYP3A4 and interaction with herbal remedies demonstrated inhibition of this hepatic enzyme by chamomile extract [21]. A study conducted in Israel evaluated the drug-herbal interactions that had occurred in patients admitted to two internal medicine departments. Of 299 patients interviewed, a potential interaction of chamomile tea with CYP3A4, that could have caused CyA elevation and toxicity due to CYP3A4 inhibition, was seen in one patient [22]. A case report has described a renal transplant patient receiving maintenance therapy with CyA (150 mg/day) and azathioprine. Initially, the patient had a CyA blood level of 180–200 ng/mL. It was discovered that the patient was drinking chamomile tea regularly and so, after discontinuing chamomile tea, the patient experienced a drop in CyA blood concentration to 50 ng/mL, while taking the same dose of CyA [23].

Given the increased CyA blood concentrations with chamomile, it is advisable that patients do not take the two concomitantly or, if they are drinking chamomile tea regularly, they should inform their physician if they plan to change or stop their tea consumption.

## 7. Liquorice

Liquorice or licorice is a sweetening agent used in foods and beverages. The sweetening effect is due to the compound glycyrrhizin. A recent study investigated the interaction of liquorice with P-gp and CYP3A4 in rats [24]. The study demonstrated that glycyrrhetic acid, a glycyrrhizin metabolite, activated P-gp and CYP3A4 function and subsequently reduced the oral bioavailability of CyA [24]. Previous studies have described a contradictory inhibition of P-gp but those results were obtained by in vitro experiments without in vivo evidence [25, 26].

Since decreased CyA bioavailability has been demonstrated in in vivo animal studies, liquorice should be avoided in patients treated with CyA.

## 8. Scutellariae Radix

Scutellariae radix is a flavonoid obtained from the root of *Scutellaria baicalensis*. It is widely used in traditional Chinese medicine to stimulate the immune system. A study in rats showed that the bioavailability of oral CyA was decreased when CyA and scutellaria were coadministered [27]. This is probably due to interactions with CYP3A4 and intestinal P-gp, but both are worthy of further investigation to improve our knowledge of interactions involving scutellaria and CYP isoenzymes and drug pump proteins. Bioavailability of intravenous CyA was not affected indicating that the interaction occurs at the absorption site [27]. Although the above mentioned study was conducted in a rat model, until human data are available to confirm or rebuff this interaction, coadministration of oral CyA and scutellaria should be avoided.

## 9. Quercetin

Quercetin is a flavonoid found in vegetables, fruits, supplements, and beverages. Higher concentrations of quercetin are found in ginkgo, onions, apples, and berries. This flavonoid has been studied for its antiviral, anti-inflammatory, apoptotic, and cytostatic properties.

An in vitro study has demonstrated noncompetitive inhibition of calcineurin by quercetin [28], suggesting that quercetin may have immunosuppressant properties and may possibly enhance the effect of CyA. However, quercetin has been found to have relevant interactions with CyA causing a reduction in CyA blood concentration; a study conducted on rats demonstrated a reduction in CyA blood concentration after oral coadministration of ginkgo and onion and oral CyA [29]. No alterations in CyA levels were found when CyA was given intravenously. In studies investigating the mechanism of inhibition in rats, quercetin glucuronides and sulfates were shown to increase CYP3A and P-gp activity, with a subsequent decrease in CyA bioavailability [30, 31]. Similar findings have been reported in pigs [32].

In another study in rats investigating the coadministration of CyA and mulberry—another fruit known to contain the antioxidant flavonoid quercetin—CyA bioavailability was significantly reduced; investigation of the mechanism showed that mulberry activation of CYP3A and P-gp was implicated [33].

In addition to its effects on CyA bioavailability, as an antioxidant, quercetin appears to protect against CyA-induced oxidative stress. Animal studies have demonstrated that quercetin (in combination with vitamin E) inhibits the hepatotoxic effects of CyA, probably due to inhibitory effects on lipid peroxidation and stimulation of hepatic catalase and glutathione peroxidase activity [34]. The ability of quercetin to ameliorate CyA-induced chronic nephrotoxicity has also been demonstrated in studies in rats [35, 36].

In view of these findings, foods or herbs rich in quercetin should be avoided in patients treated with CyA to ensure that therapeutic efficacy is maintained.

## 10. Resveratrol

Resveratrol—a polyphenol found in red wine—is a known inhibitor of CYP3A4 [37]. This is thought to be due to the lipophilicity of the polyphenol molecule [37]. Therefore, it may increase the blood concentration of CyA. Indeed, coadministration of resveratrol and CyA has been shown to enhance immune suppression [38] and should probably be avoided. Resveratrol has also been shown to exert a protective effect against CyA-induced nephrotoxicity, thought to be due to its effect on nitric oxide levels, and to be effective in modulating P-gp and CYP3A activity [39, 40].

## 11. *Serenoa repens*

*Serenoa repens* or saw palmetto is a fan palm, extracts of which are used in alternative medicine for the treatment of benign prostatic hyperplasia [41]. An in vitro study has demonstrated that *Serenoa repens* is a potent inhibitor of CYP enzymes 3A4, 2D6, and 2C9 [32]; however, a small study conducted in 12 healthy volunteers failed to demonstrate changes in CYP enzyme activity after multiple doses of *Serenoa repens* [42]. No case reports of CyA-*Serenoa repens* interaction have been published, although coadministration should probably be avoided until findings from this small study have been corroborated.

## 12. *Echinacea*

Extract or juice of *Echinacea*, a daisy-like plant, is widely used in medicine for its immune-modulatory properties. The species commonly used in medical preparations are *E. purpurea, E. pallida, *and* E. angustifolia.*

Several studies have been performed to investigate the possible interaction between this extract and CYP3A4, but with conflicting data. In vitro studies have reported variable inhibition of the CYP enzyme by *Echinacea* [21, 32], while in vivo studies have failed to demonstrate changes in CYP activation or inhibition [43, 44]. To date, no cases of altered CyA bioavailability due to *Echinacea* have been reported. However, concomitant use of *Echinacea* and CyA should be avoided until further studies have ruled out any possible interaction.

## 13. Berberine

Berberine, a bioactive herbal extract isolated from the roots and bark of *Berberis aristata* or *Coptis chinensis*, is a component of traditional Chinese medicines. Berberine is a CYP3A4 inhibitor and a P-gp transporter substrate, and interaction studies have shown that coadministration of CyA markedly elevates the blood concentration of CyA [45–48]. The increase in CyA bioavailability may be partly due to decreased metabolism because of CYP3A4 inhibition and partly due to increased uptake from the gut due to competition for the P-gp transporter [45]. In rats, berberine has been shown to be processed through hepatobiliary excretion, and its efflux appears to be influenced by P-gp coadministration [49].

In view of these findings, concomitant use of medicine containing berberine must be avoided during CyA treatment.

## 14. Conclusions

In recent years, the herbal market has been growing rapidly in Europe and America. Given the increased potential for concomitant use of herbs and traditional drugs, we require a better knowledge of the impact of potential interactions on their metabolism and bioavailability, in particular for those traditional drugs with a narrow therapeutic window such as CyA.

Various studies, in vivo and in vitro, have analyzed changes in CyA bioavailability during coadministration of herbs or herbal extracts.

Potentially clinically relevant decreases in CyA blood concentrations have been amply demonstrated for SJW in several case reports, and for ginger, liquorice, scutellaria radix, and quercetin in in vivo animal studies. On the other hand, increasing CyA concentrations have been described in patients concomitantly taking grapefruit juice, chamomile, or berberine, and in animal studies with cannabidiol and resveratrol. Discordant results have been reported from in vivo and in vitro studies concerning *Echinacea* and *Serenoa repens*, although their association with CyA should be avoided till future studies have clarified these results.

Although findings from animal studies cannot be directly translated into human subjects, they are an indicator of a potential interaction and, therefore, it would be prudent to avoid concomitant use of these herbal extracts until human clinical studies have ruled out any possible interaction and potential for reduced therapeutic effect (in the case of decreased CyA blood concentration) or toxicity (in the case of increased concentration).

In conclusion, we suggest that physicians interview their patients carefully about their use of herbs and herbal supplements before CyA administration. Moreover, patients already receiving CyA treatment should be warned about the possible interactions between herbal preparations and CyA and the potential outcomes.

## Figures and Tables

**Table 1:** A summary of commonly used herbal extracts, effect of herbal extract-cyclosporine A (CyA) interaction, and the mechanisms involved if known.

Herbal supplement or extract	Effect of interaction on CyA bioavailability	Mechanism of interaction	Studies
St John's wort	Decreased	Hypericin: P-gp Hyperforin: induces intestinal and hepatic CYP3A4, CYP2B6, and P-gp	Human
Grapefruit juice	Increased (oral only)	Inhibits intestinal CYP3A4	Human
Ginger	Decreased (oral only)	Reduces gastrointestinal motility	Animal
Cannabidiol	Increased	Inhibits hepatic CYP3A4	Animal
Chamomile	Increased	Inhibits CYP3A4	Human (case report)
Liquorice	Decreased (oral only)	Induced P-gp and CYP3A4	Animal
Scutellariae radix	Decreased (oral only)	Induces CYP3A4 and intestinal P-gp	Animal
Quercetin	Decreased (oral only)	Induces CYP3A4 and intestinal P-gp	Animal
Resveratrol	Increased	Induces CYP3A4	Animal
*Serenoa repens *	Increased (speculative)	Potent inhibitor of CYP3A4, 2D6, and 2C9	In-vitro
*Echinacea *	Increased (speculative)	Possible inhibitor of CYP (data inconclusive)	In-vitro
Berberine	Increased	Inhibits CYP3A4 and intestinal P-gp	Human
